# Influence of Pathogen Type on Neonatal Sepsis Biomarkers

**DOI:** 10.1155/2021/1009231

**Published:** 2021-11-19

**Authors:** Lyudmila Akhmaltdinova, Svetlana Kolesnichenko, Alyona Lavrinenko, Irina Kadyrova, Olga Avdienko, Lyudmila Panibratec

**Affiliations:** ^1^Karaganda Medical University, Karaganda, Kazakhstan; ^2^Regional Clinical Hospital of Karaganda, Perinatal Center No. 2, Karaganda, Kazakhstan

## Abstract

Understanding immunoregulation in newborns can help to determine the pathophysiology of neonatal sepsis and will contribute to improve the diagnosis, prognosis, and treatment and remains an urgent and unmet medical need to understand hyperinflammation or hypoinflammation associated with sepsis in newborns. This study included infants (up to 4 days old). The “sepsis” criteria was a positive blood culture. C-reactive protein demonstrates a strong dependence on the pathogen etiology. Therefore, its diagnostic odds ratio in Gram-positive bacteremia was 2.7 and the sensitivity was 45%, while Gram-negative was 15.0 and 81.8%, respectively. A neutrophil-lymphocyte ratio above 1 and thrombocytopenia below 50^*∗*^10^9^ cells/L generally do not depend on the type of pathogen and have a specificity of 95%; however, the sensitivity of these markers is low. nCD64 demonstrated good analytical performance and was equally discriminated in both Gram (+) and Gram (−) cultures. The sensitivity was 87.5–89%, and the specificity was 65%. The HLA-DR and programmed cell death protein study found that activation-deactivation processes in systemic infection is different at points of application depending on the type of pathogen: Gram-positive infections showed various ways of activation of monocytes (by reducing suppressive signals) and lymphocytes (an increase in activation signals), and Gram-negative pathogens were most commonly involved in suppressing monocytic activation. Thus, the difference in the bacteremia model can partially explain the problems with the high variability of immunologic markers in neonatal sepsis.

## 1. Introduction

Sepsis of newborns is one of the most important issues in pediatrics, the third leading cause of death in the neonatal period. Mortality rates range from 13% to 70% [1]. In Kazakhstan, the mortality rate from sepsis among children under one year of age increased to 4.3 in 2019, and in the Karaganda region, it was 8.68 per 1000 live births [2]. However, little progress has been done in the treatment of neonatal sepsis in the last three decades. Early diagnosis is crucial in the prevention of negative outcomes. However, an urgent and unsatisfied medical need for the diagnosis of sepsis-related hyperinflammation in newborns remains.

Traditionally, sepsis has been categorized as a manifestation of hyperinflammatory syndrome, but recent evidence has shown that the pathogenesis of inflammation in systemic infection is more complex. There is a shred of increasing evidence supporting the role of immunosuppression in sepsis [3, 4]. However, its role in neonatal sepsis remains to be elucidated. The unique physiological characteristics of organs and systems in the first days of life, especially the unique state of the immune system at the moment of birth, complicate the understanding of the norm and pathology in the immune regulation of newborns.

Programmed cell death-1/programmed death-ligand 1 (PD-1/PDL-1) is one of the key models in the development of sepsis-mediated immunosuppression, but its role in newborns is still poorly described [5, 6]. The study of immune suppression in neonates may be instrumental in better defining the immune pathophysiology of neonatal sepsis.

This could also aid in the identification of unique biomarkers that may have clinical relevance for immunomonitoring, predicting outcomes, or even targeted therapeutic agents. In the case of neonatal sepsis, the situation is complicated by many factors affecting the outcome and prognosis of sepsis, a wide variability in the degree of maturity at birth, dependence on weight and gestational age, which change every day of the calendar age [7]. Probably, the etiology of the pathogen plays an important role in immunoregulation.

Our research is devoted to studying the role of causative agents in proinflammatory and immunosuppressive signals of sepsis at different links of the immune response and an attempt to use them as biomarkers.

## 2. Materials and Methods

### 2.1. Patient Characteristics

This prospective controlled trial enrolled infants (up to 4 days old) in the Intensive Care Units of Regional Perinatal Center of Karaganda Research permission from the Karaganda Medical University Bioethics Committee No. 19 from 05.08.2019. Informed written consent was obtained from a parent prior to study enrolment. The criterion for determining a case of “sepsis” was a positive blood culture. The control group consisted of children who received treatment in the intensive care unit with negative blood cultures and unconfirmed infectious complications at the time of discharge.

Exclusion criteria were as follows:

1. Patients born to HIV-positive mothers
2. Patients receiving therapy with high doses of glucocorticosteroids
3. Primary immunodeficiency state
4. Blood loss
5. Severe malformations
6. Acute hemolytic disease of the newborn
7. Refusal of the patient's parents or legal representative to participate in the study

### 2.2. Bacteriological Research Methods

The analysis was carried out using the BD BACTEC™ FX (Peds Plus Medium) system. After the appearance of signs of growth, the broth was inoculated on the blood agar plate. Microorganisms were identified by using time-of-flight mass spectrometry (Microflex-LT, Bruker Daltonics). The causative agents of sepsis were divided into 2 groups according to the type of cell wall: (1) Gram-positive (Gram (+)): *Staphylococcus haemolyticus*, *Staphylococcus aureus*, *Staphylococcus epidermidis*, *Streptococcus agalactiae*, *Enterococcus faecalis*, and *Enterococcus faecium*; (2) Gram-negative (Gram (−)): *Klebsiella pneumoniae*, *Escherichia coli*, and *Enterobacter cloacae*. The etiological structure of neonatal sepsis was previously described [8].

### 2.3. Immunological Research Methods

Blood cell counting was conducted using a Mindray hematology analyzer. Blood samples were fixed with a no-wash fixation and lysis technology using OptiLyse C, no-wash lysing solution, according to the manufacturer's instructions. Surface staining for activation markers was performed with *α*CD4, *α*CD8, *α*CD14, *α*CD24, *α*CD64, and *α*CD279 (Becton Dickinson). The immunological parameters were studied with flow cytometry (Partec CyFlow Space). An unstained sample was used as a negative control. Compensation settings were made using built-in software (FlowMax). The research was carried out while standardizing the gain settings for the entire research period.

The leukocyte population was identified according to the expected size and granularity on a forward and side scatter plot (FSC/SSC), and CD24+ (neutrophils), CD14+ (monocytes), CD4+ (T-helper lymphocytes), and CD8+ (T-cytotoxic lymphocytes) were gated and defined by the characteristic phenotypes. *α*CD279 was used for the PD-1 marker.

Then, the CD64 index was defined as the ratio of the mean fluorescence intensity (MFI) of CD64+ neutrophils to the MFI of CD64 lymphocytes (internal negative control) according to the gating strategy.

Neutrophil-lymphocyte ratio (NLR) was defined as the ratio of the percentage of neutrophils to lymphocytes and the platelet-lymphocyte ratio (PLR) as the ratio of platelets to lymphocytes (expressed as 10^9^ cells/L).

The analysis of C-reactive protein (CRP) was carried out by the hospital laboratory. The analysis of the CRP data included samples, the immunological examination of which could not be done due to a deficiency of biomaterial or a clot; in general, they corresponded to the groups given in Table 1.

### 2.4. Statistical Analysis

Statistical analysis was carried out in the R statistics (compare groups and rstatix packages) and Statistica programs using the nonparametric Kruskal–Wallis test (nonparametric one-way ANOVA). For repeated pairwise comparisons, the Mann–Whitney test with Holm's correction (R statistics) was used. Intergroup comparisons with *p* values are presented in tables, and the pairwise group comparisons with individual *p* values are discussed in the text. Categorical data were calculated using the chi-square test. The parameters for cutoff were chosen empirically and according to literature data. The parameters for cutoff were chosen empirically and according to literature data.

## 3. Results

CRP is a basic indicator for assessing the activity of the inflammatory process. We present the data of its content in the groups (Table 2).

The data on the content of leukocytes and subpopulations are given in Table 3.

Taken together, these data demonstrate that the sepsis caused by Gram-positive bacteria is a less stimulus for the production of CRP, the data for this group are more variable, and although, the median values do not differ. The significance of differences from the control is achieved only in the case of Gram-negative bacteremia (control vs. Gram (−), *p*=0.04). When assessing the diagnostic significance, this assumption was confirmed.

There was neither predominant leukocytosis nor leukopenia in both sepsis groups (Table 3). Noting the absolute and relative change of subpopulations expected during the infectious process, with the difference in the median value to a greater extent between the Gram-positive bacteremia group vs. the control group (lymphocytes (%), *p*=0.03; lymphocytes (absolute count), *p*=0.013; neutrophils (%, absolute count), *p*=0.003), there is no difference between Gram-negative vs. control groups and Gram-negative vs. Gram-positive bacteremia groups.

The median NLR shows the best distinguishing ability of this indicator for Gram-positive sepsis (Gram (+) vs. control, *p*=0.004; Gram (−) vs. control, *p*=0.2). As a diagnostic tool with a cutoff of more than 1, it would be noted that with Gram-positive bacteremia, its diagnostic power is higher (DOR 24 vs. 12.6). However, it is statistically applicable in both groups of sepsis (control vs. Gram (+), *p*=0.002; control vs. Gram (−), *p*=0.03).

The platelet count is one of the important clinical and laboratory indicators of neonatal sepsis. There were observed fluctuations in the median platelet count, especially in the PLR index, but there was no statistical significance. Moreover, indicator such as thrombocytopenia less than 50 is an indicator of the septic process in newborns in both groups of sepsis (control vs. Gram (+), *p*=0.002; control vs. Gram (−) *p*=0.03). Activation markers and classic markers of sepsis are given in Table 4.

We found no difference in CD24 expression in all groups.

The expression of *α*CD64 both as MFI clearly and unambiguously changes in newborns with sepsis in comparison to the control group. The CD64 index showed the significant difference in both groups with positive culture (MFI: control vs. Gram (+), *p*=0.01; control vs. Gram (−), *p*=0.02; CD64 index: control vs. Gram (+), *p*=0.001; control vs. Gram (−), *p*=0.04; Gram (+) vs. Gram (−), *p* > 0.05).

When using a cutoff (index more 4 or MFI more 10) in both cases, good resolution is achievable (MFI: control vs. Gram (+), *p*=0.02, and control vs. with Gram (−), *p*=0.006; CD64 index control vs. Gram (+), *p*=0.002, and control vs. Gram (−), *p*=0.006). Taken together, these data demonstrate the satisfactory operating characteristic analysis of the test, which probably does not depend on the type of pathogen.

Furthermore, there were no changes in the value of HLA-DR + monocytes and lymphocytes but were observed multidirectional changes in their expression; in both cases, the difference was significant not only with the control but also with the other group of sepsis. In the case that expression on monocytes decreases with a Gram-negative pathogen (Gram (−) vs. control, *p*=0.03, and Gram (−) vs. Gram (+), *p*=0.03), then on lymphocytes, it increases with a Gram-positive pathogen (Gram (+) vs. control, *p*=0.034, and Gram (+) vs. Gram (−), *p*=0.02).

CD4+CD25+T cells level did not change significantly in any of the groups. PD-1 changes on lymphocytes and monocytes are given in Table 5.

Our findings in the study of the PD-1 receptor were partly unexpected. We found that PD-1 expression on monocytes and lymphocytes differs fundamentally depending on the type of pathogen. Thus, for PD-1 on monocytes, MFI decreases with the Gram (+) pathogen group, and the difference between the two groups (but not with the control) is significant (*p*=0.007). In contrast, the number of PD-1 on lymphocytes decreases with a Gram-negative infection, but not with a Gram-positive one. The difference between the two sepsis groups was again significant (control vs. Gram (−), *p*=0.06; Gram (+) vs. Gram (−), *p*=0.01).

## 4. Discussion

Neonatal sepsis is still a pediatric problem. The clinical manifestations of the inflammatory syndrome in newborns can be effaced or variable. The normal range of laboratory markers depends on gestational or postnatal age and fluctuates in response to coexisting noninfectious processes. Although positive culture is the gold standard, the reduced volume of blood, which applies to infants and low susceptibility to pathogen concentrations, reduces the sensitivity of the method.

Even when compared to children, newborns exhibit a unique immune response to systemic infection, making diagnosis and prognosis difficult. Thus, neonatal-specific clinical trials are needed to improve survival and long-term outcomes for these populations. A better understanding of the pathophysiology of the interaction between the infant's immune system and a pathogen will open up new opportunities to improve outcomes.

CRP is a basic diagnostic test and is most commonly used to diagnose conditions associated with hyperinflammation. Studies evaluating the role of CRP in the diagnosis of early onset neonatal sepsis (EOS) report varying sensitivity and specificity from 29% to 100% and 60% to 100%, respectively [3]. Most authors report a low clinical benefit in the case of EOS [9, 10]. There are many conditions in which there is a false increase in CRP levels. In this study, using a cutoff of 5 mg/l, we showed that such a large spread of data is probably associated with the pathogen. The ratio of the CRP level with Gram-negative showed the sensitivity twice higher, and it is 81.8% versus 45% with Gram-positive bacteremia, and the total DOR is 2.7 vs. 15.0, while the NPV is 0.9% versus 64.5% and while the PPV is 60%.

Standard blood test parameters were used for the diagnosis of EOS, but without much success. According to some authors, leukopenia has shown low sensitivity (29%) but high specificity (91%) for the diagnosis of neonatal sepsis [11]. Sharma et al. [7] claimed that values under 5000/mm^3^ for WBCs have a high specificity (91%) regarding sepsis diagnosis. While according to Philip A.G., neutropenia was more predictable for neonatal sepsis than neutrophilia [12]. In this study, the changes in the number of neutrophils and lymphocytes are in response to systemic infection; however, the discriminant ability was poor and unlikely to be clinically useful apart.

There have already been studies showing that NLR has better diagnostic capabilities than CRP, including in premature and low birth weight babies [13–15]. However, there remains the issue of cutoff, which is noticeably different for each author and should change dynamically with each calendar day. It was suggested that using the NLR, which showed a specificity of 95% DOR (12.6 and 24.4, respectively), had some etiological influences on the development of sepsis. We found the best diagnostic capabilities of this parameter in Gram-positive infection.

Thrombocytopenia is one of the markers of neonatal sepsis in children associated with negative outcomes [16]. Although, not all authors agree that this indicator is useful in diagnosis and prognosis [17, 18]. In this study, thrombocytopenia was closely associated with sepsis, and although the sensitivity did not exceed 40%, the specificity was 95%. PLR is an index that did not show any significance.

Markers of neutrophil and monocyte activation have long been used as markers of sepsis. nCD64 has been the most promising marker for neonatal sepsis and is still on the rise. A meta-analysis investigating the use of nCD64 as a biomarker of NS that included 17 studies with 3,478 participants revealed only a modest pooled sensitivity of 0.77 (95% CI 0.74–0.79), specificity of 0.74 (95% CI 0.72–0.75), and AUC of 0.87 [19–21]. This study demonstrated nCD64 as a universal marker, approximately equally reflecting both Gram-negative and Gram-positive bacteremia, without difference in absolute or relative terms, with a maximum sensitivity of 90% and a specificity of 65%.

The expression of HLA-DR is one of the first cytometric markers of sepsis, the most popular, but it is more commonly used in late neonatal sepsis and much more successfully in adults. It reflects the anergy of the immune system as a consequence of systemic inflammation and is associated with negative outcomes [22, 23]. There were no significant differences in the number of HLA-DR + monocytes and lymphocytes; however, the expression of HLA-DR on monocytes was significantly reduced in the group with Gram-negative bacteremia. On the other hand, the opposite tendency was registered on lymphocytes, with Gram-positive systemic infection, and the expression was higher in both the control group and in the group with Gram-negative sepsis.

PD-1 is an immune checkpoint molecule, which plays an important role in downregulating the immune system's proinflammatory activity. The new paradigm of sepsis suggests that immunosuppression is part of the immunopathology in systemic infection, and PD-1 activation plays a key role. Using adult patients as examples, most authors show that PD-1 increases with severe systemic infection [4]. But its role is not well understood in the immune response of newborns, and there are only scattered studies. Zasada et al. [5] confirmed the generally accepted change in PD-1 expression in late neonatal sepsis, an increase in the marker of immune depletion on monocytes in the severe course and negative outcome. Unexpectedly, there was no increase in PD-1 in the study groups. In the case that amount of PD-1 on monocytes decreased during Gram-positive systemic infection, the number of PD-1 + lymphocytes (CD4+ and CD8+ T cells) would decrease during Gram-negative infection. This can be explained by the specifics of the early neonatal period, in which the development of tolerance to environmental antigens and the inflammatory response compete with each other and the outcome will depend on the ratio of these processes. Moreover, a notable fact is that it was not singled out critical and shocked patients, and most of the studied children experienced sepsis.

## 5. Conclusions

This study demonstrated that the difference in the pattern of bacteremia may partially explain the problems with the wide variability of immunological markers in neonatal sepsis. Although the basic popular markers of innate immunity (NLR, thrombocytopenia, and CD64) can be equally applied in early neonatal sepsis of various etiologies with satisfactory operational characteristics, CRP is largely stimulated by Gram-negative pathogens.

In addition, some combined shifts in activation and suppression markers specific to different types of the pathogen were revealed; thus, monocytes are probably more sensitive to the tolerogenic effect of endotoxin, and the expression of the antigen-presenting receptor decreases, whereas lymphocytes receive either an increase in the activating signal (Gram-positive bacteria) or a decrease in the suppressive signal (Gram-negative pathogen), and any case, the proinflammatory response predominates over immunosuppression.

The study of the possibility of quantifying the expression of various markers by MFI has not yet exhausted its potential; however, such studies require methodological support and opportunities for standardization.

## Figures and Tables

**Table 1:** Patient information.

Parameter	Control	Gram-positive bacteremia	Gram-negative bacteremia	*P* value
Birth weight (g), Me (Q1; Q3)	2175 (1708; 2771)	2060 (1335; 2925)	2575 (1440; 2951)	>0.05
Gestational age (week), Me (Q1; Q3)	34 (33; 37)	33 (29.5; 37)	33.5 (31.5; 36.25)	>0.05
Cesarean section, Me (Q1; Q3)	11/20 (55%)	11/16 (68%)	5/9 (55%)	>0.05
*n* (for CRP)	26	20	11	—
*n* (for biomarker)	20	16	10	
*P* value, Kruskal–Wallis test for comparing 3 groups.				

**Table 2:** CRP data in the study groups.

Parameter	Control	Gram-positive bacteremia	Gram-negative bacteremia	*P* value
CRP (mg/l), Me (Q1; Q3)	0.6 (0.0; 4.5)	5.3 (0.0; 6.0)	6.0 (5.6; 12.0)	**0.02**
CRP >5 mg/l (count/all)	6/26 (23%)	9/20 (45%)	9/11 (82%)	**0.004**
Diagnostic odds ratio		2.7 (0.76; 9.6)	15.0 (2.52; 89.2)	
DOR (±95% CI)		45	81.8	
Sensitivity, %		76.9	76.9	
Specificity, %		60	60	
Positive predictive value (PPV), %		64.5	90.9	
Negative predictive value (NPV), %				
Statistically significant *p* values are in bold.				
*P* value, Kruskal–Wallis test for comparing 3 groups.				

**Table 3:** The main indicators of leukocytes and platelets.

Parameter	Control	Gram-positive bacteremia	Gram-negative bacteremia	*P* value
Leukocytosis (10^9^ cells/l), Me (Q1; Q3)	18.3 (14.4; 20.7)	13.3 (12.3; 15.1)	15.3 (8.8; 25.2)	>0.05
Leukopenia less than 5^*∗*^10^9^ cells/l	1/20 (5%)	1/16 (6.25%)	1/10 (10%)	>0.05
Leukocytosis more than 20^*∗*^10^9^ cells/l	7/20 (35%)	3/16 (18.75%)	3/10 (30%)	>0.05
Lymphocytes (%), Me (Q1; Q3)	52.0 (44.9; 68.0)	40.0 (32.3; 51.1)	49.3 (35.6; 54.1)	**0.03**
Neutrophils (%), Me (Q1; Q3)	29.0 (19.2; 40.8)	47.1(37.5; 57.1)	39.5 (34.4; 43.2)	**0.003**
Lymphocytes (10^9^ cells/l), Me (Q1; Q3)	10.0 (7.2; 13.1)	5.0 (3.5; 6.7)	6.4 (3.1; 14.3)	**0.02**
Neutrophils (10^9^ cells/l), Me (Q1; Q3)	4.55 (3.1; 6.3)	6.0 (4.0; 8.8)	4.9 (2.43; 10.0)	>0.05
NLR, Me (Q1; Q3)	0.56 (0.27; 0.80)	1.39 (0.72; 1.52)	0.82 (0.63; 1.3)	**0.006**
NLR>1 (count/all)	1/20 (5.0%)	9/16 (56.3%)	4/10 (40%)	**0.004**
DOR (±95% CI)		24.4 (2.6; 229)	12.6 (1.17; 136)	
Sensitivity, %		52.6	40	
Specificity, %		95	95	
Positive predictive value (PPV), %		90.0	80	
Negative predictive value (NPV), %		73.0	76	
Platelets (10^9^ cells/l), Me (Q1; Q3)	151.0 (131.0; 167.0)	116.0 (50.0; 176.0)	154 (47.0; 176.0)	>0.05
PLR, Me (Q1; Q3)	16.62 (10.1; 19.8)	25.9 (12.4; 39.1)	12.4 (10.1; 12.56)	>0.05
Platelets less 50 10^9^ cells/l (count/all)	1/20 (5%)	6/16 (37.5%)	4/10 (40.0%)	**0.031**
DOR (±95% CI)		11.4 (1.2; 108)	12.6 (1.1; 136)	
Sensitivity, %		37.5	40	
Specificity, %		95	95	
Positive predictive value (PPV), %		85.7	80	
Negative predictive value (NPV), %		62.5	76	
Statistically significant *p* values are in bold.				
*P* value, Kruskal–Wallis test for comparing 3 groups.				

**Table 4:** Activation markers.

Parameter	Control	Gram-positive bacteremia	Gram-negative bacteremia	*P* value
MFI nCD24, Me (Q1; Q3)	9.9 (7.48; 14.1)	7.16 (4.44; 15.12)	8.02 (7.48; 20.9)	>0.05
MFI nCD64, Me (Q1; Q3)	8.37 (5.0; 13.0)	15.3 (10.8; 24.7)	15.5 (12.5; 17.8)	**0.003**
MFI nCD64 > 10 (count/all)	7/20 (35%)	12/16 (75%)	9/10 (90%)	**0.004**
DOR (±95% CI)		5.5 (1.3; 23.9)	16.7 (1.7; 160)	
Sensitivity, %		75	90	
Specificity, %		65	65	
Positive predictive value (PPV), %		63.2	56.3	
Negative predictive value (NPV), %		76.5	92.9	
Index CD64, Me (Q1; Q3)	3.07 (1.94; 6.3)	11.05 (7.7; 19.45)	7.2 (6.7; 10.4)	**0.002**
Index CD64 > 4 (count/all)	7/20 (35%)	14/16 (88%)	9/10 (90%)	**<0.001**
DOR		13.0 (2.2; 74.3)	16.7 (1.7; 160)	
Sensitivity, %		87.5	90	
Specificity, %		65	65	
Positive predictive value (PPV), %		66.6	56.3	
Negative predictive value (NPV), %		86.6	92.86	
HLA-DR + Mon, Me (Q1; Q3)	95 (79.0; 98.0)	88.0 (80; 98)	88 (75.0; 90.0)	>0.05
MFI monHLA-DR, Me (Q1; Q3)	3.59 (2.38; 7.5)	5.2 (2.59; 6.7)	2.1 (1.63; 2.9)	**0.015**
HLA-DR + lymph, Me (Q1; Q3)	5.7 (4.5; 8.6)	5.16 (3.0; 6.6)	9.82 (3.72; 16.94)	>0.05
MFI lymphHLA-DR, Me (Q1; Q3)	9.7 (8.2; 23.0)	16.1 (13.7; 17.7)	11.6 (6.7; 12.5)	**0.008**
CD25 + CD4+, Me (Q1; Q3)	16.0 (6.96; 14.75)	25.5 (10.0; 55.00)	17.0 (12.25; 38.25)	>0.05
Statistically significant *p* values are in bold.				
*P* value, Kruskal–Wallis test for comparing 3 groups.				

**Table 5:** PD-1 on lymphocytes and monocytes.

Parameter	Control	Gram-positive bacteremia	Gram-negative bacteremia	*P* value
PD-1 + Mon	37.2 (33.0; 43.9)	37.5 (33.0; 40.0)	44 (38.0; 45.5)	0.15
PD-1 + Mon MFI	25.7 (13.4; 33.8)	15.4 (14.7; 18.0)	31 (20.0; 39.8)	**0.008**
PD-1 + CD4	30 (21.3; 36.5)	34.3 (33.0; 42.0)	13.5 (6.5; 33.0)	**0.0035**
PD-1 + CD4 MFI	13.7 (9.3; 18.3)	18.0 (12.6; 26.6)	13.0 (12.0; 14.0)	0.3
PD-1 + CD8	41.5 (40.0; 44.5)	42.0 (41.5; 45.5)	37.0 (34.0; 39.0)	**0.003**
PD-1 + CD8 MFI	16.2 (12.0; 17.8)	15.2 (14.9; 28.0)	12.2 (10.0; 13.6)	0.208
Statistically significant *p* values are in bold.				
*P* value, Kruskal–Wallis test for comparing 3 groups.				

## Data Availability

The data used to support the findings of this study are available at https://doi.org/10.6084/m9.figshare.15132027 (https://figshare.com/).
